# Design of an Optical Device Based on Kirigami Approach

**DOI:** 10.3390/ma17051211

**Published:** 2024-03-06

**Authors:** Marta De Giorgi

**Affiliations:** Department of Engineering for Innovation, University of Salento, 73100 Lecce, Italy; marta.degiorgi@unisalento.it

**Keywords:** kirigami, rotating squares, camouflage, transparency, color changing

## Abstract

The aim of this work was to design a kirigami-based metamaterial with optical properties. This idea came from the necessity of a study that can improve common camouflage techniques to yield a product that is cheap, light, and easy to manufacture and assemble. The author investigated the possibility of exploiting a rotation to achieve transparency and color changing. One of the most important examples of a kirigami structure is a geometry based on rotating squares, which is a one-degree-of-freedom mechanism. In this study, light polarization and birefringence were exploited to obtain transparency and color-changing properties using two polarizers and common cellophane tape. These elements were assembled with a rotating-square structure that allowed the rotation of a polarizer placed on the structure with respect to a fixed polarizer equipped with cellophane layers.

## 1. Introduction

The aim of this project was to design a mechanical metamaterial that can exploit the optical properties of easily accessible commercial devices. The idea came from the necessity of a study that can improve common camouflage techniques to make a product more marketable in numerous fields, such as robotics and defense, due to it being cheaper, lighter, and easier to manufacture and assemble. Nature serves as a remarkable source of inspiration for camouflage techniques, which are widely utilized by diverse species, such as cephalopods, chameleons, and other animals, for protection from predators. In contemporary military and civilian contexts, the goal of camouflage technology is to obscure the presence of objects and make them virtually undetectable to potential threats. This field of research has long captivated scientists who have sought to manipulate different physical phenomena, including optics, electromagnetics, acoustics, and thermotics. By understanding an object’s unique scattering signature across various physical fields, it becomes possible to perceive and identify it. Drawing parallels to the phenomenon of wave-dynamic illusion, optical camouflage has the potential to alter actual perceptions and create controlled illusions, thereby enabling groundbreaking applications in the realms of optical cloaking and illusion. In nature, camouflage is achieved through the incorporation of a diverse range of colors, vivid geometric and random patterns, and morphological structures that aid animals in evading detection by predators. The most commonly observed strategies include background matching, disruptive coloration, and disguise. Background matching involves animals possessing colors and patterns that closely resemble their surrounding habitats. By blending in with their environment, they become less conspicuous to predators. Disruptive coloration, on the other hand, relies on contrasting patterns that break up the silhouette of an animal, making it more difficult for predators to discern its true shape. This technique helps to confuse and deceive potential threats. In the disguise strategy, an organism takes on the shape and coloration of the plants or animals in its immediate vicinity. By mimicking its surroundings, it becomes camouflaged and harder to detect.

A mechanical metamaterial is an artificial material that has properties that are well known in nature but are difficult to detect. These properties are attributable to its geometry, which has a key role in this kind of material and makes these assets completely tunable, changing characteristic parameters. A variety of applications have been demonstrated for metamaterials, and metamaterials are going to be commercialized soon. As mentioned, geometry is the core of a mechanical metamaterial: the geometry has a key role in its mechanical properties in terms of the displacement of the structure. The final geometry is chosen by taking into consideration the final output from the whole system. From a classical point of view, materials engineering involves determining how to modify the internal chemical composition of materials and their external properties using supplementary treatments that could be helpful in changing the response of the material. This confirms that metamaterials have changed research. Metamaterials have applications in all of the following fields: acoustics [1] (sound absorption), bioengineering [2,3] (design of metamaterials or meta-surfaces of phantoms for surgical use), soft robots [4,5] (design of soft robots based on metamaterial structures; development of tunable, programmable, and tethered locomotion), optics [6] (metamaterials for cloaking), electromagnetism [7] (resonators and antennas), automotive industry [8] (design of airbags and MEMS sensors), sports [9] (shoe support through assistive grips), thermal expansion [10] (tunable metamaterials for managing thermal expansion), and vibration absorption [11]. There are a lot of geometries that can be exploited for a wide range of purposes. Some examples are origami and kirigami. Origami is an ancient Japanese art. The etymology of the word comes from “oru” and “kami”, which mean, respectively, “to fold” and “paper”. In this art, structures are obtained by folding simple paper to obtain certain geometries. Kirigami is a Japanese art as well. The word’s etymology comes from “kiru” and “kami”, which mean “to cut” and “paper”, respectively. Starting from this, we can understand that the main difference between the two methods is the way of obtaining geometries, by either folding or cutting [12]. Aesthetically, origami consists of very pleasant patterns and shapes that have applications in deployable structures, medical stents for cardiovascular surgery, flexible electronic devices, and the reproduction of space satellites. The art of origami also has some specific techniques that are useful in engineering. It is important to underline that this type of metamaterial is based on a one-degree-of-freedom mechanism. This makes it tunable, mathematically manageable, and programmable in terms of mechanical response (forces or displacement). Kirigami metamaterials are obtained with a series of cuts into a thin sheet, such as plastic shims. The responses of kirigami metamaterials are tunable as well through the dimensions of these cuts, which become the key design parameter. This material also exhibits an out-of-plane deformation [13]. This property has been studied and largely applied to engineering, for example, for text encryption [14,15]. Text encryption is helpful for hiding messages starting from flat sheets that in undeformed conditions seem to be normal-colored sheets but, once stretched in a chosen direction, can generate some message or image [16]. Another important application is the use of metamaterials in internal design engineering, such as windows that can be designed using the kiri-kirigami technique actuated through remote thermal actuation with the aim of reflecting and redirecting the sunlight to save energy [14]. One of the most important examples is a geometry based on rotating squares [12], which is a one-degree-of-freedom mechanism. This structure represents a simple solution for the aim of this work: the possibility of exploiting a rotation to produce transparency and color changing. In this study, two important optical properties, polarization and birefringence, have been used to accomplish our goals. By incorporating rotation or dislocation, a system or object can exhibit the ability to switch between different functionalities or states. This additional freedom allows for dynamic changes in the system’s configuration, leading to versatile and adaptive behaviors. By leveraging rotation or dislocation as additional degrees of freedom, engineers and researchers can design systems that possess enhanced versatility, adaptability, and the capability to switch between different functionalities [17,18]. For color changing to occur, a relative rotation between two polarizers is utilized here. This means that one polarizer is fixed, and the other is movable. Attached to the fixed polarizer is one or more layers of cellophane tape. This is the key element that allows the color changing to take place; in fact, the cellophane tape is the main component responsible for changes in the birefringence index in the whole system that was implemented in this study. Once all the project’s main characteristics were chosen, we implemented a mathematical model to characterize the polarizers and, after building a color database, we tried to set the model in order to predict color changing.

## 2. Materials and Methods

**Figure 1:**
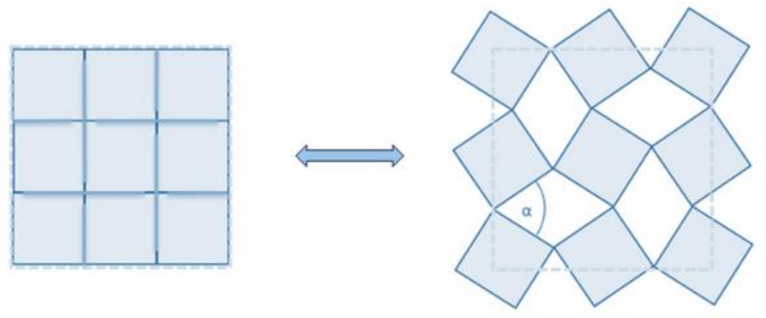
Rotating-square mechanism [12].

In this study, the geometry that characterizes the kirigami-based material is based on the “rotating-square mechanism”. The rotating-square mechanism is a single-degree-of-freedom mechanism exhibiting an auxetic behavior. In this structure, rigid squares are connected at nodes through simple hinges. Under an external load, the squares start rotating at the vertices, expanding or contracting in the orthogonal direction with respect to the load depending on the loading type and starting condition, if open or closed (Figure 1). The first researchers to explore this kind of material were Grima et al. [12].

The fusion of mechanical metamaterials and optical properties represents the innovative aspect of this project. The key point is the transparency of the material placed between two polarizers, as it allows for reaching the most vivid color.

The steps that characterized this project are summarized as follows:

- Choice of the system geometry: rotating squares (first choice because a rotation is required for exploiting optical properties);
- Rotating squares’ manufacturing using an acrylic sheet (first choice);
- Rotating squares’ manufacturing using polydimethylsiloxane (PDMS, flexible manufacturing, second choice) to reach higher transparency;
- Cellotape application on the fixed polarizer to obtain color changing.

### 2.1. Light Modulation

A polarizer can be seen as a black box with a specific transfer function that allows only a specific light to pass through it [19]. If another layer of polarizer is put on top of the first one, in the case of linear polarizers, one specific direction of polarization can be observed. The direction of the electric field that can cross the polarizer is the main direction of polarization. The angle between the two sheets determines how much light can pass through the polarizer; the output will be a reduction in the light intensity. If polarizers are aligned, the output will be transparent. If they are crossed, no light will cross, and the output will be opaque. So, by rotating a polarizer and fixing the other one, it is possible to reach a variable transparency. A characterization consists in evaluating how much light passes a specific position of the two polarizers at specific angles. A MatLab code was developed for characterizing polarizers based on Jones matrices, and its results were compared with experimental ones. From the experimental side, the characterization consisted of the following steps: record a movie while rotating the polarizers from 0° to 90°, import the recorded movie in MatLab, and track the recorded movie with a script. The designed script captured a single video frame and took the color of the polarizers’ overlapped area in RGB scale (red, green, blue scale: a scale that describes all colors through a digital mixing of the three main colors). The script converted the color in RGB to greyscale (a scale based on the intensity of the grey) with the function “RGBtoGreyscale”; in this way, it is easy to obtain the intensity of light as an output. Then, the transparency value was computed (Equation (1)) in comparison to the total opaque value:

(1) $$Transparency={[(I}_{i}-I_{total\;opaque})/I_{i}]$$

where *I_i_* is the intensity of light in input.

### 2.2. Birefringence: The Physical Property Used for Obtaining Colors

**Figure 2:**
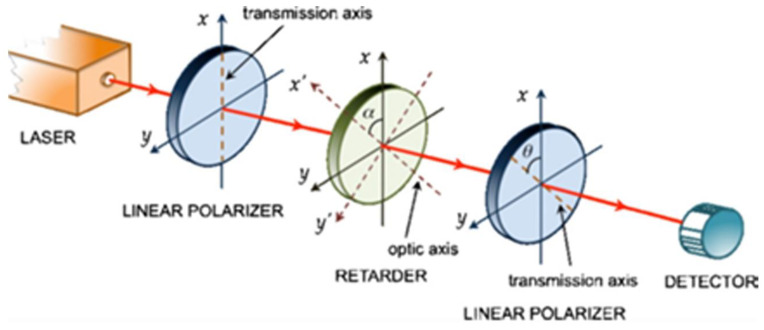
Example of experimental setup for observing birefringence [20].

Birefringence was discovered in 1669 by Erasmus Bartholinus (1625–1698) using calcite [19]. Birefringence is a synonym of double refraction. Refraction is the phenomenon where the direction of a wave is altered as it moves from one medium to another, caused by its change in speed. This phenomenon occurs in all the waves present in nature, and it is controlled by a parameter named the refraction index. Refraction occurs if two objects have two different refraction index values. Birefringence occurs in anisotropic elements. In Figure 2, an experimental setup made up of a light source, two linear polarizers, and a “retarder” between the two linear polarizers is shown [20]. A retarder is an optical device that modifies the polarization state of a light wave as it passes through. It is typically constructed using a birefringent material, which has different refractive index values for light polarized along two specific perpendicular crystal axes. When light passes through the retarder, its polarization state is altered based on the differential phase delay experienced by the two perpendicular polarization components. This modification can be used for various applications, such as controlling the polarization of light or creating optical effects [21]. The incident light that comes out from the first polarizer changes the phase angle according to Equation (2). Birefringence can be used to produce the desired phase angle to obtain some new results that could be based on deviation or on a lower wavelength as an output.

(2) $$\gamma =\frac{2\pi \left(∆n\right)\;*\;d}{\lambda_{0}}$$

where *γ* is the phase difference between the orthogonally polarized waves introduced by the birefringent plate of thickness *d*, ∆*n* is the difference in refraction, *λ*_0_ = wavelength.

In the developed experiments, the role of the retarder was played by common cellophane tape. Cellotape exhibits birefringence due to its manufacturing process. When cellophane is stretched during production, the organic molecules within it align and straighten in the direction of the stretch. This alignment causes a difference in the dielectric constant and subsequently the refractive index along the direction of the tape compared to the perpendicular direction. These two directions serve as principal axes, and as a result, the polarization components parallel and perpendicular to the length of the tape travel at different velocities. This differential velocity leads to the phenomenon of birefringence in cellotape [22,23,24]. The cellotape was placed between two polarizers and illuminated with a light source of a given temperature (5000 K) on the background.

### 2.3. Manufacturing of Rotating Squares

Two possible configurations were explored: rigid squares, where the involved material was acrylic, and flexible squares, where the involved material was polydimethylsiloxane (PDMS). Rigid squares were manufactured with laser-cut acrylic frames. The laser cutter used was the UNIVERSAL PLS6.150D, a CO_2_ laser with a power of 150 W and 0.3 mm as best precision supplied by Universal Laser Systems, Inc., Scottsdale, AZ, USA [25].

The manufacturing process was composed of the following steps:

- Set the position of the acrylic sheet on the honeycomb table;
- Set the height precisely in order to have the maximum laser power on the cutting surface, close the top, and set the cut parameters (power: 70%, speed: 5%);
- Cut the frame starting from a CAD drawing previously saved as a .dxf file;
- Wait 30 s and open the top of the machine;
- Obtain the final item, and in the case of an additional undesired part, remove it;
- Obtain a plastic sheet (a blue one has been chosen for our purposes with a 0.90 mm thickness) and put it on the honeycomb table;
- Set the height again;
- Cut the design for the hinges (power: 7%, speed: 20%);
- Obtain the final item and remove all the undesired parts.

Once all the profiles were cut, all the squares were cleaned under water with soap to obtain maximum transparency. Once the squares were dry, it was easy to complete the final assembly, which consisted of locating and gluing the hinges inside the cut slot using small clamps used for surgical purposes.

## 3. Results

### 3.1. Light Modulation

**Figure 3:**
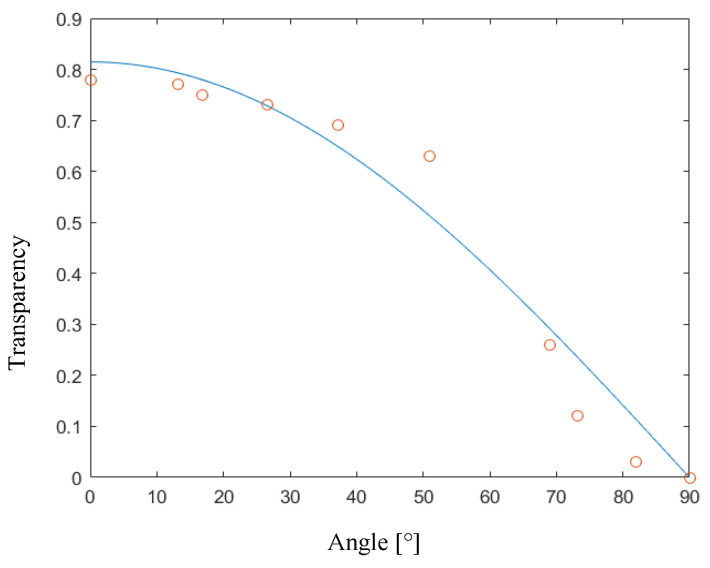
Characterization of a polarizer. Transparency vs. angle between the polarizers. Experimental data (orange circles) vs. mathematical model output (blue line).

The implemented MatLab script provided the interesting results shown in Figure 3, where the transparency (Equation (1)) predicted by the script (blue line) and the experimental data captured through video tracking (orange circles) are reported.

### 3.2. Birefringence: The Physical Property Used for Obtaining Colors

**Figure 4:**
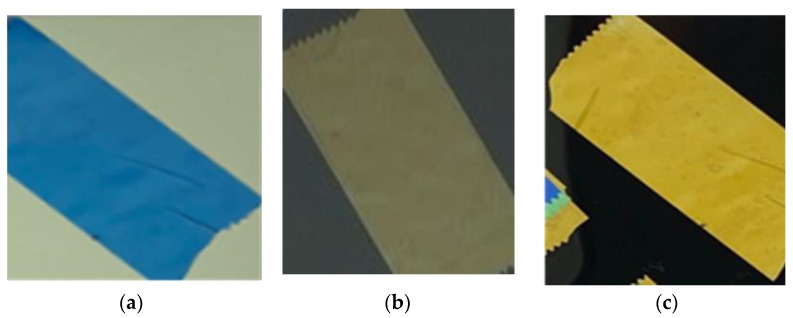
Color output from cellotape between two polarizers at 0° (**a**), 45° (**b**), and at 90° (**c**).

The output obtained by the setup shown in Figure 2 was a vivid color that came out from the tape. The colors shown in Figure 4a were obtained by orienting at 0° the main polarization direction of the two polarizers and using only one layer of tape. When one of the two polarizers was rotated 45° and 90°, the color changed from blue to light yellow to gold yellow (visible in Figure 4b,c). By adding more layers of tape at different angles, more colors could be reached. A good result was obtained with two layers of tape, where a color change was clearly visible, but with three layers of tape, the obtained results were really interesting. A color database (Table 1) was built by using three layers of tape and rotating the movable polarizer up to 180°. Three different setups were assembled, and colors were captured frame by frame in RGB (red, green, blue digital scale) with MatLab code. The setups used were composed of three layers of tape mounted with the following angles: first setup: 0°, 20°, 45°; second setup: 0°, 35°, 80°; third setup: 100°, 130°, 190°.

Mathematically, birefringence is a phenomenon that can be described through Jones matrices [20]. A MatLab code based on Jones matrices was developed to predict colors.

**Figure 5:**
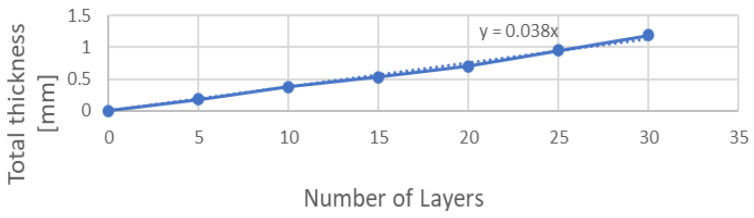
Measurement of cellophane tape thickness.

**Figure 6:**
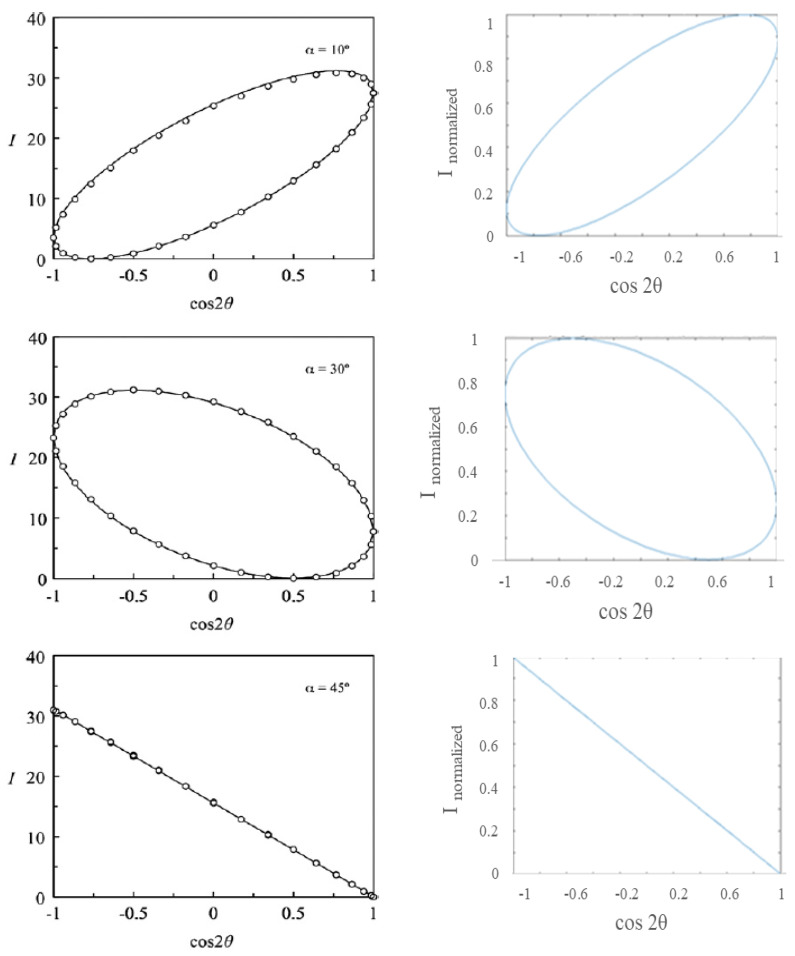
Validation of the mathematical model: intensity of light vs. cosine of the angle between polarizers. **Left column**: results from [20]. **Right column**: output obtained from the developed mathematical model for different orientations of tape (10°, 30°, 45°).

The first step was to consider all the setups shown in Table 1. Each of these setups can be considered as a black box with an input. There is a wavelength that is dominating and that is producing a color output. The goal was to find it. The key step was to find the normalized output after determining the birefringence index. The birefringence index (Δ*n*) was evaluated from the inverse formula of phase shift *γ* (Equation (2)) for a fixed wavelength [20]. The tape thickness was measured by assembling several lay-ups made up of 5, 10, 15, 20, and 25 layers of tape and measuring each lay-up five times. The searched thickness was the slope of the line in Figure 5: 0.038 mm. Then, the birefringence index was computed through an iteration, and it was equal to 0.0077. The mathematical model was qualitatively validated, as shown in Figure 6, by comparing its results with experimental data found in literature [20]. The graphs show the trend of the light intensity with respect to the cosine of the angle between the polarizers for different tape orientations. Note that the output of the MatLab model was normalized, differently from the model developed in [20] which provided an intensity of light module as an output.

**Figure 7:**
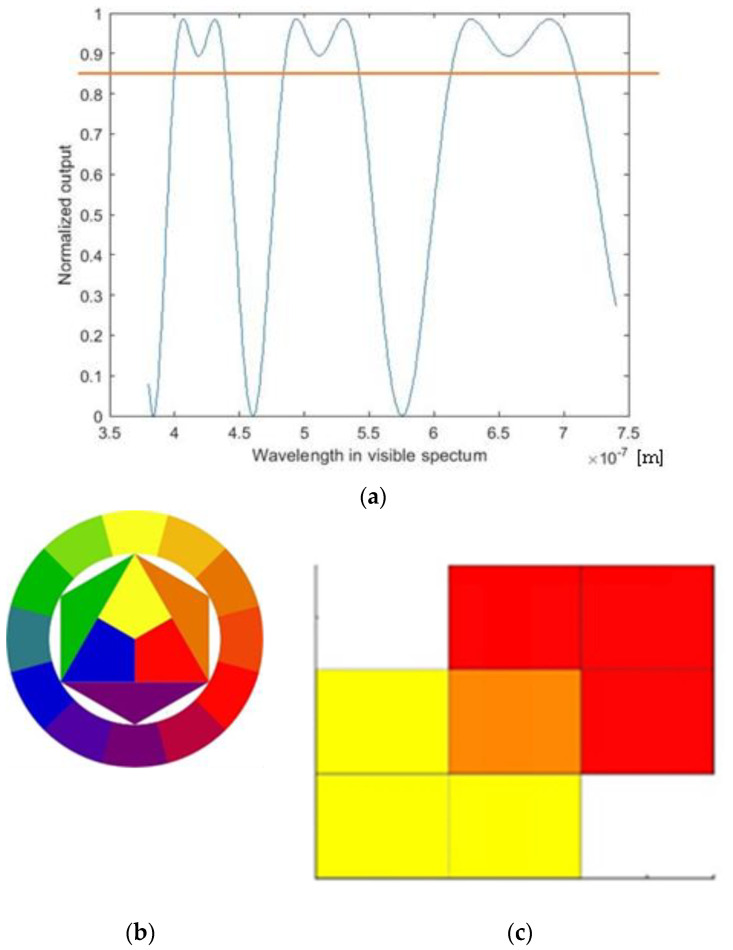
(**a**) Model output for a specific angle between polarizers: Normalized output vs. wavelength. The orange line represents the threshold imposed for defining which of the wavelengths is dominant. (**b**) Hitten circle. (**c**) Overlapping of patches—mixing color.

Then, the output should be the colors reached for specific angles between polarizers and for specific angles of tape assembly. The best results were reached in the following way: Split the visible spectrum into ranges that identify colors according to the theory of colors; Identify the wavelengths at which the value of I_normalized_ overtakes a threshold value; these are the dominant wavelengths identified with an orange line in Figure 7a, i.e., the wavelengths that participate in the final color. Create patches of the colors identified in the previous step through the dominant wavelengths. Obtain the final color according to the Hitten circle [22] (Figure 7b,c).

**Figure 8:**
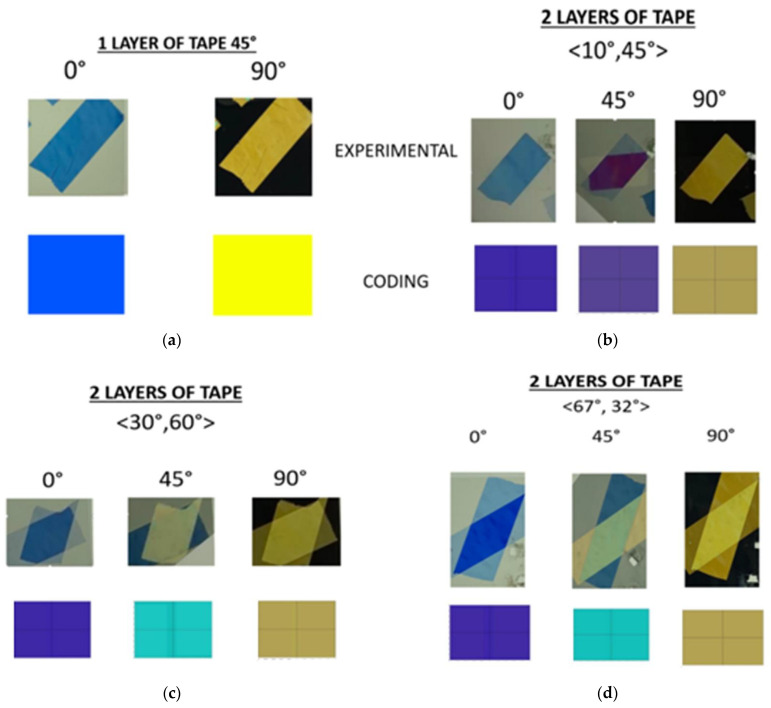
Results from color prediction: (**a**) an example with 1 layer of tape; (**b**–**d**) examples with 2 layers of tape example at different angles: <10°, 45°>, <30°, 60°>, <67°, 32°>, respectively.

A comparison between the experimental and predicted results is shown in Figure 8.

Slight differences between the real and predicted colors are due to the discretization of the model. In fact, the developed MatLab code tried to find a solution to a physical problem in a rigorous mathematical way. Another problem is that it is really challenging to have a precise conversion from a wavelength obtained as output to a specific RGB color; this happens because a specific wavelength does not correspond to one and only one RGB color. The relationship between wavelengths and RGB colors is not straightforward. Converting a specific wavelength to an RGB color involves mapping that wavelength onto the RGB color space. However, this mapping is not a one-to-one relation because the RGB color space cannot represent all possible spectral colors. The RGB color space has a limited gamut, and some colors within the visible spectrum may fall outside this gamut. Therefore, the conversion from wavelength to RGB color involves approximation and may involve interpolation or other mathematical techniques for estimating the closest RGB representation for a given wavelength. The accuracy of the conversion depends on the specific algorithm or mapping used and the limitations of the RGB color space [26,27,28]. On the other hand, the process of spectral color rendering takes into account the human visual system’s response to different wavelengths of light. Human eyes have three types of color receptors, often referred to as cones, which are most sensitive to different regions of the visible spectrum. These cones are sensitive to short, medium, and long wavelengths and are commonly associated with blue, green, and red light, respectively. Factors such as lighting conditions, light source, individual differences in color perception, and color context can influence how colors are perceived. Moreover, RGB is a device-dependent color model: different devices detect or reproduce a given RGB value differently since the color elements (such as phosphors or dyes) and their response to the individual red, green, and blue levels vary from manufacturer to manufacturer, or even in the same device over time. As a consequence, the comparison (Figure 8) between predicted and real or, more precisely, perceived color necessarily involves discrepancies.

### 3.3. Manufacturing of Rotating Squares

**Figure 9:**
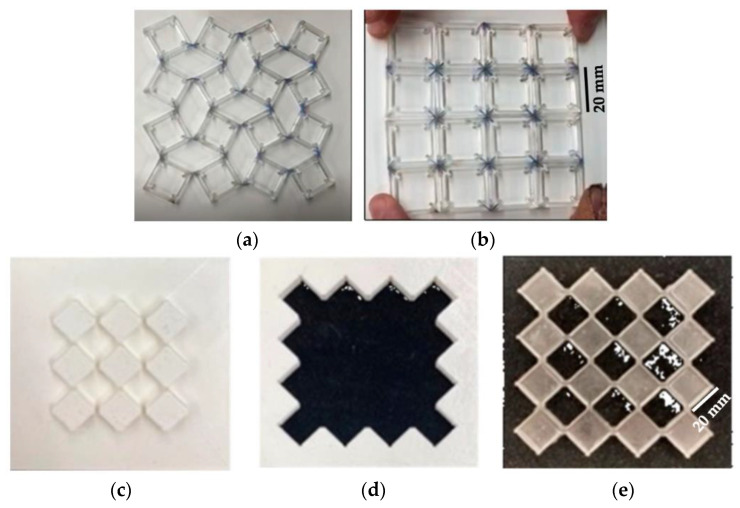
Final assembly of acrylic rotating square 10 mm thick: open (**a**) and closed (**b**) position; PDMS rotating squares 10 mm thick: 3D-printed mold parts (**c**,**d**) and demolded structure (**e**).

The final result of the assembly process illustrated in Section 2.3 is shown in Figure 9 in open (Figure 9a) and closed positions (Figure 9b). Figure 9c–e show 3D-printed mold parts and a demolded structure, respectively.

**Figure 10:**
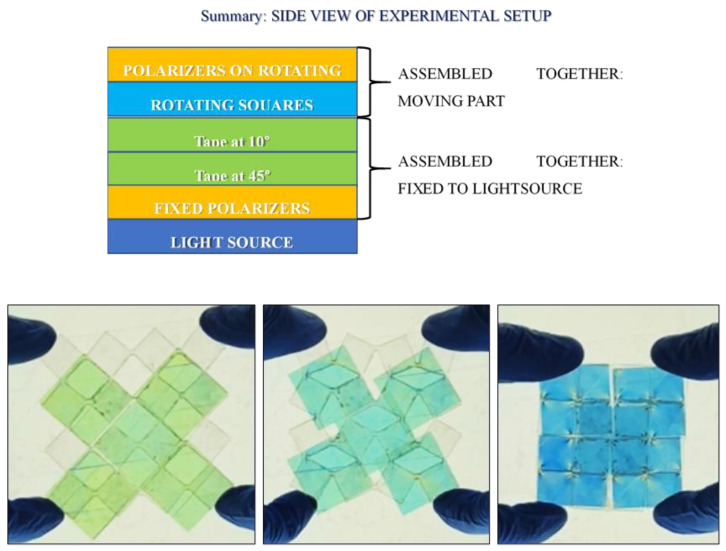
Experimental setup and experimental results: 0% strain (open); transitory state; 100% strain (closed).

The evident limit of this structure was transparency. Acrylic sheets were obtained from MacMaster-Carr, IL, USA; according to the technical datasheet, they have 92% transparency. Since the final goal of this project was to obtain color changing using the rotation between two polarizers and a birefringent tape in the middle, the rotating element had to have as high as possible transparency to obtain a good final result, i.e., vivid colors. As a result of this need, the material was replaced with the PDMS. Sylgard 184 from Dow Corning, MI, USA, was chosen due to its popularity in biomedical and microelectronic applications. The two components were mixed with a 10:1 mixing ratio, which is recommended by the seller Dow for obtaining the best properties, and cured at room temperature for 48 h [29]. PDMS is a hyperelastic material, known for its bioengineering and microelectronic applications due to its capability of penetrating microscopic holes. PDMS is an inorganic polymer based on a silica–oxygen chain. Its most important properties are its high transparency and biocompatibility; it is also inert, non-toxic, and non-flammable. Rotating squares were obtained by pouring PDMS into a 3D-printed mold. The Ultimaker 3D-printer and PLA were used. Once the mold was produced, the solution was mixed using a “Thinky” mixer. In this machine, mixing was performed for 2 min at 2000 RPM, followed by defoaming for 6 min at 2200 RPM. At the end of this process, the solution was clear and free of bubbles, which is important for obtaining higher transparency. The manufacturing results are shown in Figure 9. A lay-up composed of the layers reported in Figure 10 with two layers of tape mounted at 45° and at 10° provided the results shown in Figure 10. In that figure, three reached colors, namely green, cyan, and blue, are clearly visible. This means that rotation of structures like rotating squares can be exploited for obtaining color and that the project aim was reached successfully: optical properties and mechanical metamaterials were coupled to obtain color changing, text encryption, and light modulation. Using the structure shown in Figure 9 as a basic unit, it is possible to build a display as an arrangement of several unit cells with different cellotape layers set up to obtain different colors with the same rotation. Based on this principle, it is also possible to realize text encryption with the appropriate positioning of cellotape.

## 4. Conclusions

An optical device for achieving color changing was designed. To develop this project, rotation between a fixed polarizer and a movable one was fundamental. Rotating squares were used to obtain rotation. They were manufactured with two different materials: laser-cut acrylic and plastic sheets (to reproduce the hinges) and polydimethylsiloxane (PDMS), which was used to reach higher transparency.

Two polarizers were used to modulate the passing light. Cellophane tape, which gives birefringence to the system, was mounted on the fixed polarizer. It has been demonstrated that only two vivid colors were obtained with one layer of tape. When tape layers were added, more colors were obtained due to a rotation between the two polarizers. A color database was built with three tape layers in three different setups. The color changing was visible and clear.

A MatLab code based on Jones matrices was implemented to predict colors. The implementation of the code involved defining the Jones matrices for the specific optical elements involved in the system, such as polarizers, retarders, or waveplates. The code then performed calculations using matrix multiplication to determine the polarization state of the light after it passed through the optical elements. By comparing the predicted colors from the MatLab code with the experimental observations, a good agreement was observed. This indicates that the code accurately modeled the behavior of light and provided reliable predictions for the resulting colors.

A lot of applications can be related to this project. The color changing reached is linked to only one geometry, but by building a sheet made up of a lot of connected unit cells, a wide range of colors can be reached, and text encryption can be obtained. Moreover, this kind of setup can be reproduced to obtain a wide range of colors for obtaining camouflage. Hence, all the applications related to the manipulation of light could be implemented; for example, some devices can be designed to obtain a color change in a room or just to modulate the quantity of incoming light in an area.

## Figures and Tables

**Table 1:** Color database for 3 different setups.

Polarizer Angle	R	G	B	Color Output	R	G	B	Color Output	R	G	B	Color Output
	Setup 1 (0°, 20°, 45°)				Setup 2 (0°, 35°, 80°)				Setup 3 (100°, 130°, 190°)			
0°	40	71	100		18	48	100		85	90	84	
32°	52	25	78		38	101	106		115	114	94	
65°	87	51	25		94	125	81		99	98	68	
90°	110	100	41		115	111	48		67	67	39	
138°	86	125	99		80	40	38		33	39	53	
165°	48	97	102		42	28	87		66	71	74	
180°	43	70	97		23	47	97		82	87	81	

## Data Availability

All data generated or analyzed during this study are included in this published article.
